# Expression of TXNIP in Cancer Cells and Regulation by 1,25(OH)_2_D_3_: Is It Really the Vitamin D_3_ Upregulated Protein?

**DOI:** 10.3390/ijms19030796

**Published:** 2018-03-10

**Authors:** Mohamed A. Abu el Maaty, Fadi Almouhanna, Stefan Wölfl

**Affiliations:** Institute of Pharmacy and Molecular Biotechnology, Heidelberg University, Im Neuenheimer Feld 364, 69120 Heidelberg, Germany; Abdelgawad@stud.uni-heidelberg.de (M.A.A.e.M.); f.almouhanna@stud.uni-heidelberg.de (F.A.)

**Keywords:** vitamin D, TXNIP, VDUP1, cancer

## Abstract

Thioredoxin-interacting protein (TXNIP) was originally identified in HL-60 cells as the vitamin D_3_ upregulated protein 1, and is now known to be involved in diverse cellular processes, such as maintenance of glucose homeostasis, redox balance, and apoptosis. Besides the initial characterization, little is known about if and how 1,25-dihydroxyvitamin D_3_ [1,25(OH)_2_D_3_] induces TXNIP expression. We therefore screened multiple cancerous cell lines of different tissue origins, and observed induction, repression, or no change in TXNIP expression in response to 1,25(OH)_2_D_3_. In-depth analyses on HL-60 cells revealed a rapid and transient increase in TXNIP mRNA levels by 1,25(OH)_2_D_3_ (3–24 h), followed by a clear reduction at later time points. Furthermore, a strong induction in protein levels was observed only after 96 h of 1,25(OH)_2_D_3_ treatment. Induction of TXNIP expression by 1,25(OH)_2_D_3_ was found to be dependent on the availability of glucose in the culture medium, as well as the presence of a functional glucose transport system, indicating an inter-dependence of 1,25(OH)_2_D_3_ actions and glucose-sensing mechanisms. Moreover, the inhibition of de novo protein synthesis by cycloheximide reduced TXNIP half-life in 24 h, but not in 96 h-1,25(OH)_2_D_3_-treated HL-60 cells, demonstrating a possible influence of 1,25(OH)_2_D_3_ on TXNIP stability in long-term treatment.

## 1. Introduction

1,25-dihydroxyvitamin D_3_ [1,25(OH)_2_D_3_] is the biologically active form of vitamin D_3_, and is the natural ligand of the nuclear vitamin D receptor (VDR) [1]. Upon binding to its receptor, 1,25(OH)_2_D_3_ induces immense changes in gene expression patterns in different cells, with several genes identified as either direct—harboring vitamin D response elements (VDRE)—or indirect targets of the molecule. Such genes include those involved in the regulation of proliferation, such as p21 and p27, apoptosis-related genes, like BAX, and tissue-specific differentiation genes, such as prostate specific antigen [1,2,3]. Among the putative vitamin D target genes is thioredoxin-interacting protein (TXNIP), which is located on chromosome 1q21.1. 

TXNIP was originally reported by Chen and Deluca in 1994 as the VDUP1 (vitamin D_3_ upregulated protein 1) [4]. Their search of 1,25(OH)_2_D_3_-responsive cDNAs in HL-60 (acute myeloid leukemia) cells led to the identification of this gene, whose mRNA levels were found to be induced by 1,25(OH)_2_D_3_ treatment in as early as 6 h, plateaued at 18 h, and was maintained till 24 h. In 1999, Nishiyama and coworkers reported that VDUP1 is the binding partner of reduced thioredoxin, and thus acts as its negative regulator through inhibiting its anti-oxidant function [5]. Since then, a plethora of publications from different groups has shown that, in addition to influencing redox homeostasis, TXNIP senses intracellular levels of glucose and glycolytic intermediates, as well as levels of adenosine-containing molecules [6,7,8]. In response to an increase in intracellular glucose levels, the heterodimer MondoA:MLX (Max-like protein X) translocates to the nucleus, and binds to carbohydrate response elements (ChoRE) on the promoter of the *TXNIP* gene, inducing its expression [6]. TXNIP in turn works to reduce intracellular glucose levels, by decreasing its uptake, possibly through limiting the membrane availability of glucose transporter 1 [9]. 

With regards to cancer, TXNIP is proposed to be a potential tumor suppressor due to its ability to induce oxidative stress-mediated apoptosis, as well as due to its reduced expression and silencing in tumor tissues and cancer cell lines [10,11]. Therefore, compounds that reactivate TXNIP expression are viewed as promising anti-tumor candidates, such as the histone deacetylase inhibitor suberoylanilide hydroxamic acid [12] and the histone methyltransferase inhibitor 3-Deazaneplanocin A [13]. Consequently, due to the “historical” association between 1,25(OH)_2_D_3_ and TXNIP as a vitamin D upregulated protein, it has been suggested that 1,25(OH)_2_D_3_ could be used to reactivate TXNIP in cancers [10]. To our knowledge, this association has only been shown in HL-60 cells [14], and thus extrapolation of this relationship to other cancers is highly speculative. 

Recently, we showed that in the prostate cancer cell line LNCaP, 1,25(OH)_2_D_3_ treatment induces diverse metabolic changes that lead to a significant reduction of TXNIP levels [15]. This non-canonical regulation prompted us to investigate whether 1,25(OH)_2_D_3_ is capable of inducing TXNIP expression in different cancer types, including those with silenced TXNIP expression. An initial screen of different cancer cell lines that are treated with 1,25(OH)_2_D_3_ surprisingly showed all the possible options, induction, reduction, and no change in TXNIP levels in response to treatment. A detailed analysis of 1,25(OH)_2_D_3_ treatment in HL-60 cells, in which the up-regulation was initially discovered, demonstrated a transient increase in TXNIP mRNA levels in response to treatment that was diminished at later time points. Despite the reduced TXNIP mRNA levels upon longer term treatment, TXNIP protein levels were clearly higher after 1,25(OH)_2_D_3_ treatment compared to cells treated with dimethyl sulfoxide (DMSO). Inhibition of de novo protein synthesis in 1,25(OH)_2_D_3_-treated cells by cycloheximide (CHX) did not reduce TXNIP protein levels at a time point when mRNA levels were reduced (96 h), suggesting an increased TXNIP half-life in the presence of 1,25(OH)_2_D_3_. Additionally, we observed a lack of TXNIP induction on the mRNA and protein levels, as well as a lack of oxidative stress in response to 1,25(OH)_2_D_3_ treatment in the absence of glucose, hinting at a critical cross-talk between VDR mediated gene regulation and glucose-sensing transcriptional machinery like MondoA/MLX.

## 2. Results

### 2.1. 1,25(OH)_2_D_3_ Induces, Reduces and Has No Effect on TXNIP Levels in Cancer Cells of Different Tissue Origins

**Figure 1:**
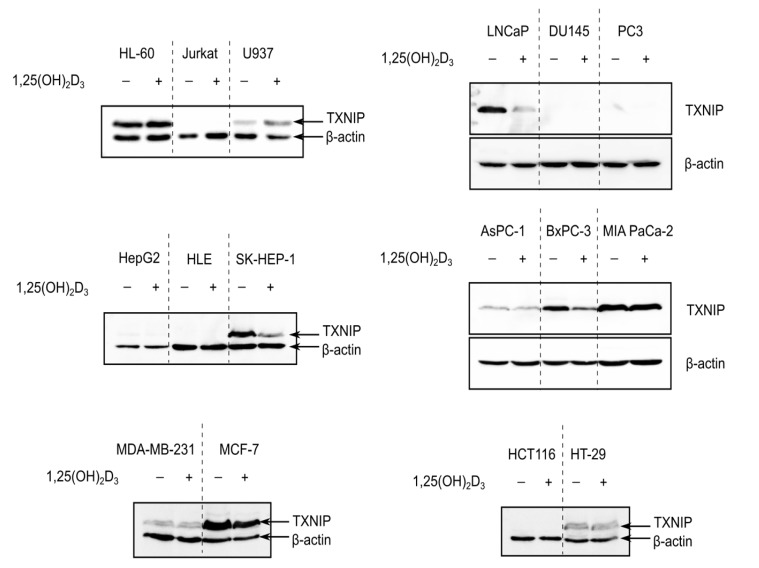
TXNIP is differentially regulated by 1,25(OH)_2_D_3_ in cancer cells. Immunoblots showing TXNIP protein levels in cancer cells of various tissue origins—hematological (HL-60, Jurkat, U937), prostate (LNCaP, DU145, PC3), liver (HepG2, HLE, SK-HEP-1), pancreatic (AsPC-1, BxPC-3, MIA PaCa-2), breast (MDA-MB-231, MCF-7), and colorectal (HCT116, HT-29)—after treatment with 100 nM 1,25(OH)_2_D_3_ for 72 h. In several cell lines, e.g., Jurkat cells, TXNIP is not detected and thus no influence of 1,25(OH)_2_D_3_ on its expression can be observed. In cases were basal levels are detected, 1,25(OH)_2_D_3_ either induced, reduced, or had no clear effect on TXNIP expression, e.g., U937, MCF-7, and HT-29, respectively. “+” and “−” denote the presence or absence of the indicated molecule/treatment, respectively.

To investigate whether 1,25(OH)_2_D_3_ is capable of inducing TXNIP expression in different cancer models, cell lines of various tissue origins—hematological, prostate, pancreatic, liver, colorectal, and breast—were treated with either DMSO or 1,25(OH)_2_D_3_ (100 nM) for 72 h and TXNIP levels were analyzed using immunoblotting. The investigated cell lines exhibited disparate basal levels of TXNIP, where some cell lines had completely repressed TXNIP expression, with the colorectal cancer cell line HCT116 as an example, some cell lines had high basal expression of the protein, like the breast cancer cell line MCF-7, and finally, cell lines with lower TXNIP levels, like the pancreatic cancer cell line AsPC-1 (Figure 1).

In cell lines where TXNIP expression was silenced, 1,25(OH)_2_D_3_ did not appear to induce the expression of the protein, whereas in cell lines that expressed TXNIP at varying degrees, 1,25(OH)_2_D_3_ exerted either the expected “classical” induction, for example in U937 (histiocytic lymphoma), or non-canonical reduction, as observed in LNCaP, BxPC-3, and MCF-7 (prostate, pancreatic, and breast cancer cells, respectively), but also had no clear effect on TXNIP levels, like in HT-29 cells (colorectal cancer) (Figure 1).

Interestingly, we did not observe a clear induction in TXNIP levels in response to 1,25(OH)_2_D_3_ in HL-60 cells (Figure 1), and thus performed an extensive analysis into the temporal regulation of TXNIP by 1,25(OH)_2_D_3_, with the aim to identify possible modulatory mechanisms adjusting the metabolic response to 1,25(OH)_2_D_3_ in HL-60 cells, thereby triggering differential TXNIP regulation, as well as to investigate the possible dependence of this regulation on glucose-related signaling.

### 2.2. 1,25(OH)_2_D_3_ Transiently Induces TXNIP mRNA Levels but Increases Protein Levels in Long-Term Treatments

**Figure 2:**
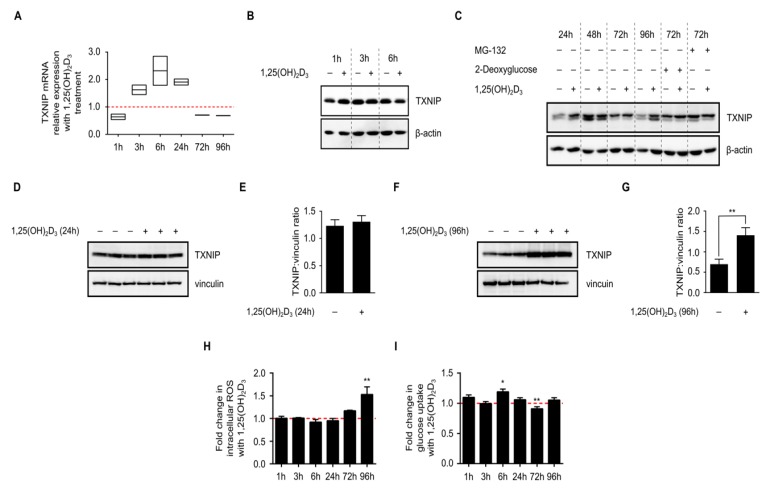
Time-dependent regulation of TXNIP expression and associated cellular processes in HL-60 cells by 1,25(OH)_2_D_3_. (**A**) 1,25(OH)_2_D_3_ (100 nM) induced a transient increase in TXNIP mRNA levels that was diminished at later time points. The dashed red line indicates the baseline TXNIP mRNA expression in DMSO-treated cells set to 1; (**B**,**C**) Immunoblot showing TXNIP protein levels in response to 1,25(OH)_2_D_3_; induction is observed after 24 h and also after 96 h of treatment. Basal TXNIP levels exhibit temporal fluctuations, independent of treatment. Treatment with 2-deoxyglucose (10 mM), a potent inducer of TXNIP expression, and MG-132 (5 µM), a proteasomal inhibitor to prevent degradation, were used as positive controls. The compounds were added into the conditioned medium of DMSO- and 1,25(OH)_2_D_3_-treated HL-60 cells either 24 h (2-deoxyglucose) or 6 h (MG-132) before the end of the initial treatment period. “+” and “−” denote the presence or absence of the indicated molecule/treatment, respectively; (**D**) Immunoblot and (**E**) densitometric quantification of TXNIP protein levels after 24 h of treatment of HL-60 cells with 1,25(OH)_2_D_3_ indicate a mild increase in TXNIP level, which lacks statistical significance; (**F**) Immunoblot and (**G**) densitometric quantification after 96 h of treatment of HL-60 cells with 1,25(OH)_2_D_3_ showing a statistically significant induction of TXNIP levels; (**H**) Intracellular ROS levels, presented as fold change of treated vs. control cells, were found to be significantly induced by 1,25(OH)_2_D_3_ only at the latest time point (96 h). The dashed red line indicates the baseline ROS level in DMSO-treated cells set to 1; (**I**) Glucose uptake, which is known to be inhibited by TXNIP, was found to be significantly induced by 1,25(OH)_2_D_3_ at an early time point (6 h), and reduced at a later one (72 h). Baseline glucose uptake level in DMSO-treated cells is indicated by the dashed red line set to 1. Statistical significance was calculated using a two-tailed Student’s *t*-test. *p*-Values less than or equal to 0.05 and 0.01, are depicted by * and **, respectively. Error bars ± SD; *n* = 3.

Since the original publication describing VDUP1 demonstrated the induction of TXNIP/VDUP1 by 1,25(OH)_2_D_3_ on the mRNA level at time points ranging from 6 to 24 h [4], we aimed to reproduce their findings, and add later time points to investigate whether their observed induction was transient or not. A time course spanning 1 to 96 h was employed and mRNA expression analysis was performed using RT-qPCR (Reverse Transcription-quantitative PCR). We observed a clear induction in TXNIP mRNA levels with 1,25(OH)_2_D_3_ treatment at time points 3, 6, and 24 h, and a surprising reduction at 72 and 96 h, demonstrating that the induction in TXNIP mRNA levels by 1,25(OH)_2_D_3_ is not sustained in the longer time treatments (Figure 2a). 

Having analyzed mRNA levels we next analyzed TXNIP protein levels in HL-60 cells upon 1,25(OH)_2_D_3_ treatment at the same time points using immunoblotting (Figure 2b,c), and observed fluctuations in the basal levels independent of treatment, which could be attributed to changes in nutrient availability in the environment, or to the natural glucose-sensing, homeostatic mechanism that TXNIP is known to influence. We measured glucose levels in the culture medium of DMSO- and 1,25(OH)_2_D_3_-treated HL-60 cells and observed that while the glucose levels were clearly reduced in the medium of DMSO-treated cells, the levels were not diminished (Figure S1), demonstrating that the relative decrease in basal TXNIP level at the latest time point is not due to glucose depletion, but rather reflects the influence of glucose homeostasis on TXNIP expression. When compared to DMSO-treated cells, and independent of temporal fluctuations in basal expression, 1,25(OH)_2_D_3_ was found to mildly induce TXNIP levels in 24 h and strongly in 96 h (Figure 2c–g).

We then investigated changes in cellular parameters known to be influenced by TXNIP status, namely intracellular reactive oxygen species (ROS) levels and glucose uptake, in response to 1,25(OH)_2_D_3_ treatment. Intracellular ROS levels were largely unaffected by the treatment across most of the investigated time points, except for the latest one (96 h), where 1,25(OH)_2_D_3_ was found to strongly and significantly induce ROS levels (Figure 2h). On the other hand, an induction in glucose uptake was observed after 6 h of treatment with 1,25(OH)_2_D_3_, an effect that was diminished in later time points (Figure 2i). In fact, after 72 h of treatment with 1,25(OH)_2_D_3_, glucose uptake was found to be clearly reduced when compared to DMSO-treated cells (Figure 2i). 

In view of the presented results, we hypothesized that: (i) glucose availability and subsequently recruitment of transcriptional machinery capable of regulating TXNIP expression, might be crucial in mediating 1,25(OH)_2_D_3_’s effect, since basal expression levels exhibited profound temporal fluctuations, reflecting the time-dependent effects of cellular glucose homeostasis on TXNIP expression, and (ii) transcriptional induction of TXNIP by 1,25(OH)_2_D_3_ may not be solely responsible for the observed upregulation on the protein level since at the latest time point, TXNIP mRNA levels were found to be reduced, whereas the protein expression induced by treatment, highlighting the possible involvement of protein stabilizing mechanisms at later time points, and finally (iii) 1,25(OH)_2_D_3_ induces changes in glucose metabolism—glycolysis and/or mitochondrial respiration—that stimulate TXNIP expression.

### 2.3. Glucose Availability Is Crucial for 1,25(OH)_2_D_3_-Mediated Regulation of TXNIP mRNA and Protein Levels as Well as Associated Oxidative Stress

**Figure 3:**
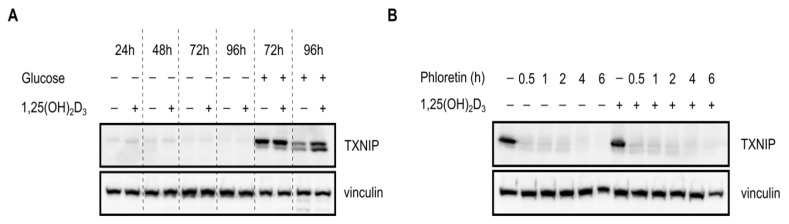

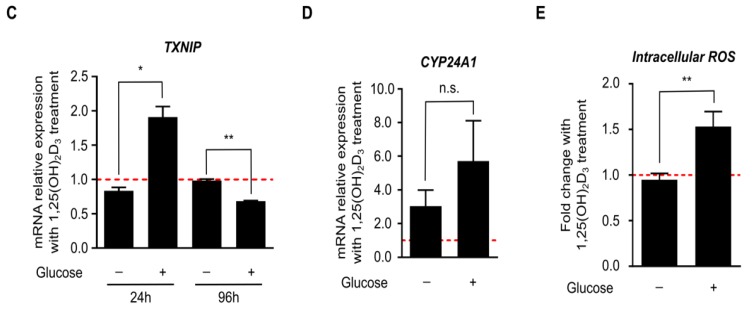
Regulation of TXNIP expression and redox balance in HL-60 cells by 1,25(OH)_2_D_3_ is glucose-dependent. (**A**,**B**) Immunoblots showing TXNIP protein levels. (**A**) In the absence of glucose, expression of TXNIP in HL-60 cells is significantly reduced, and appears to be unaffected by 1,25(OH)_2_D_3_ (100 nM) treatment. “+” and “−” denote the presence or absence of the indicated molecule/treatment, respectively; (**B**) In the presence of a single high dose of the glucose uptake inhibitor phloretin (200 µM), added at different time intervals prior to the end of the initial treatment period (24 h), 1,25(OH)_2_D_3_ is found to be incapable of inducing TXNIP expression; (**C**) Changes in TXNIP mRNA levels after 24 and 96 h of treatment with 1,25(OH)_2_D_3_ in HL-60 cells, cultured in the presence or absence of glucose, reveal that regulation by 1,25(OH)_2_D_3_ is glucose-dependent. The dashed red line indicates baseline mRNA expression in DMSO-treated cells set to 1. Error bars ± SD; *n* = 2; (**D**) Unlike TXNIP, the 1,25(OH)_2_D_3_ target gene CYP24A1, is induced in response to 1,25(OH)_2_D_3_ treatment (96 h) independent of glucose availability. Error bars ± SD; *n* = 2; (**E**) In absence of glucose, intracellular ROS levels are largely unaltered upon treatment of HL-60 cells for 96 h with 1,25(OH)_2_D_3_. The dashed red line indicates basal ROS levels in DMSO-treated cells set to 1. Statistical significance was calculated using a two-tailed Student’s t-test, with p-values less than or equal to 0.05 and 0.01 depicted by * and **, respectively. Nonsignificant results are denoted as n.s. Error bars ± SD; *n* = 3.

As previously mentioned, tight regulation of glucose homeostasis by TXNIP is orchestrated through the nuclear translocation of the heterodimer MondoA/MLX, which regulates TXNIP expression by binding to ChoRE on the gene’s promoter. Given the observed temporal fluctuations in TXNIP expression (Figure 2c), we postulated that glucose-sensing mechanisms might be involved in mediating 1,25(OH)_2_D_3_’s effects on TXNIP mRNA and protein levels. We thus cultured HL-60 cells and performed treatments for various time points in glucose-free medium, and investigated TXNIP mRNA and protein expression. In the absence of glucose, TXNIP expression was found to be clearly diminished across all the time points, and addition of 1,25(OH)_2_D_3_ was incapable of inducing its expression (Figure 3a). Similarly, in the presence of a single high dose of the glucose transporter inhibitor phloretin, TXNIP expression was not induced by 1,25(OH)_2_D_3_ (Figure 3b). Additionally, the observed regulation of TXNIP mRNA expression by 1,25(OH)_2_D_3_, whether induction at 24 h, or reduction at 96 h, was not observed in the absence of glucose (Figure 3c), unlike the clear induction in the mRNA levels of the 1,25(OH)_2_D_3_ target gene CYP24A1, which was glucose-independent (Figure 3d). Interestingly, the strong increase in oxidative stress observed at the latest time point with 1,25(OH)_2_D_3_ treatment, typically associated with elevated TXNIP levels, was not observed in the absence of glucose (Figure 3e). Altogether, we concluded that regulation of TXNIP expression and associated oxidative stress by 1,25(OH)_2_D_3_ is glucose-dependent, indicating a possible cross-talk between VDR-signaling and glucose-sensing mechanisms.

### 2.4. Induction in TXNIP Levels by 1,25(OH)_2_D_3_ Is Possibly Orchestrated through a Complex Interplay between Transcriptional Induction and Protein Stability

**Figure 4:**
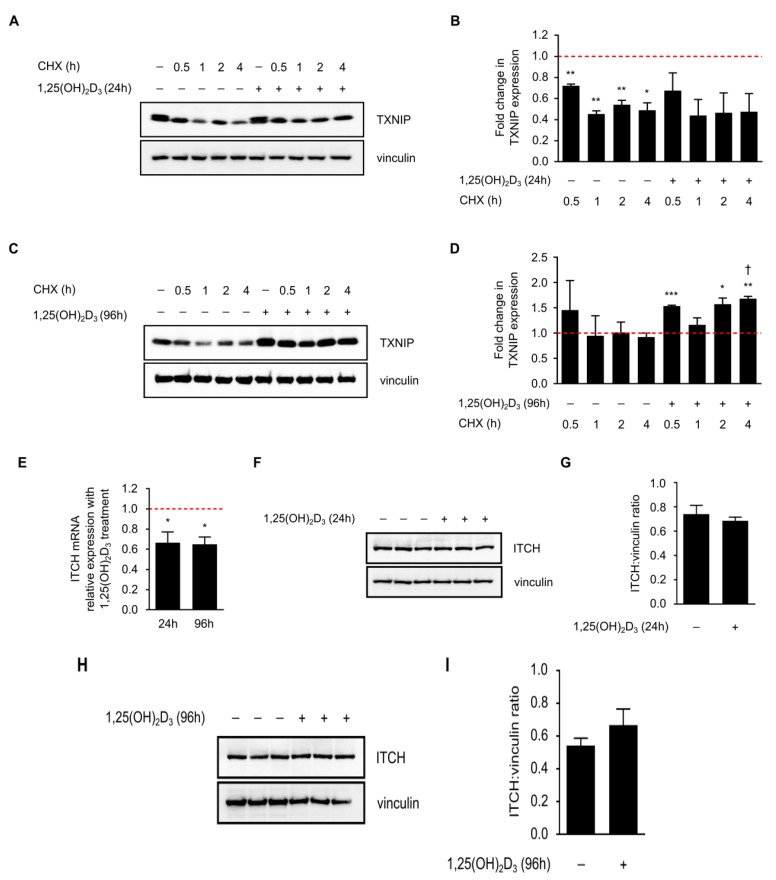
Cycloheximide (CHX) reduces TXNIP half-life in 24, but not in 96 h 1,25(OH)_2_D_3_-treated HL-60 cells. (**A**) Inhibition of protein synthesis by CHX (10 µM) treatment, added into the conditioned medium of DMSO- and 1,25(OH)_2_D_3_-treated HL-60 cells, at different time intervals prior to the end of the initial treatment period (24 h), led to reduced TXNIP protein levels in the presence and absence of 1,25(OH)_2_D_3_. “+” and “−” denote the presence or absence of the indicated molecule/treatment, respectively; (**B**) Densitometric quantification of two similar biological replicates. The dashed red line indicates baseline TXNIP protein expression in DMSO-treated cells set to 1; (**C**) In 96 h-treated HL-60 cells, CHX reduced or had no effect on TXNIP protein levels in absence or presence of 1,25(OH)_2_D_3_, respectively; (**D**) Densitometric quantification of 2 similar biological replicates. Statistical comparisons made between the different conditions and DMSO-treated cells were performed using a two-tailed Student’s *t*-test. *p*-Values less than or equal to 0.05, 0.01, and 0.001, are depicted by *, **, and ***, respectively. A dagger indicates statistical significance compared to the corresponding mono-treatment. Error bars ± SEM; (**E**) ITCH mRNA expression analysis in response to 24 and 96 h of treatment with 1,25(OH)_2_D_3_. The dashed red line indicates baseline ITCH mRNA level in DMSO-treated cells set to 1. Error bars ± SD; *n* = 2; (**F**,**G**,**H**,**I**) Analysis of ITCH protein levels after a (**F**,**G**) 24 h or (**H**,**I**) 96 h treatment with 1,25(OH)_2_D_3_; (**G**,**I**) Densitometric quantifications illustrate only mild differences in response to treatment.

We then aimed to address the second hypothesis on the possible involvement of protein stabilizing mechanisms. To confirm the lack of translational induction of TXNIP in HL-60 cells by 1,25(OH)_2_D_3_ at later time points, cycloheximide (CHX), which is an inhibitor of de novo protein synthesis, was added to 24 and 96 h DMSO- or 1,25(OH)_2_D_3_-treated HL-60 cells, for different periods (0.5, 1, 2, and 4 h before the end of the DMSO/1,25(OH)_2_D_3_ treatment period). In 24 h-treated cells, CHX treatment was found to reduce TXNIP protein half-life both in the presence and absence of 1,25(OH)_2_D_3_ (Figure 4a,b). On the other hand, in 96 h-treated cells, CHX did not hamper the induction in TXNIP levels in the presence of 1,25(OH)_2_D_3_, but moderately reduced its levels in the absence of 1,25(OH)_2_D_3_ (Figure 4c,d).

In view of this observation, we postulated that long-term treatment of HL-60 cells with 1,25(OH)_2_D_3_ induces TXNIP expression by reducing the levels of protein degradation machinery, thereby stabilizing TXNIP. It has been shown recently that the E3 ubiquitin ligase ITCH, targets TXNIP for proteasomal degradation [16]. We thus speculated that 1,25(OH)_2_D_3_ may reduce the expression of this protein. While ITCH mRNA levels were indeed found to be significantly reduced by 1,25(OH)_2_D_3_ after 24 and 96 h of treatment (Figure 4e), protein levels were insignificantly influenced by treatment across the same time points (Figure 4f–i). We therefore conclude that while ITCH regulation by 1,25(OH)_2_D_3_ may have mildly contributed to TXNIP stability, other uncharacterized factors may have also played a role, since 1,25(OH)_2_D_3_ treatment has been shown to reduce the expression of proteasome subunits in HL-60 cells, as well as modulate the expression of various genes of protein degradation machinery in other cell lines [17,18]. 

### 2.5. 1,25(OH)_2_D_3_ Does Not Influence Glucose Metabolism in HL-60 Cells but Modulates Overall Intracellular Energy Levels

Based on our previous findings in prostate cancer cells [15], we postulated that 1,25(OH)_2_D_3_ could regulate TXNIP expression in HL-60 cells by inducing metabolic changes that stimulate its expression. Such metabolic alterations may include: (i) reduction of glycolytic rate leading to a relative accumulation of glycolytic intermediates capable of inducing TXNIP levels, and (ii) induction of mitochondrial activity and thus ATP production, which could drive ATP-requiring glycolytic reactions, e.g., that catalyzed by hexokinase, thereby increasing the levels of glycolytic intermediates and subsequently TXNIP expression. 

**Figure 5:**
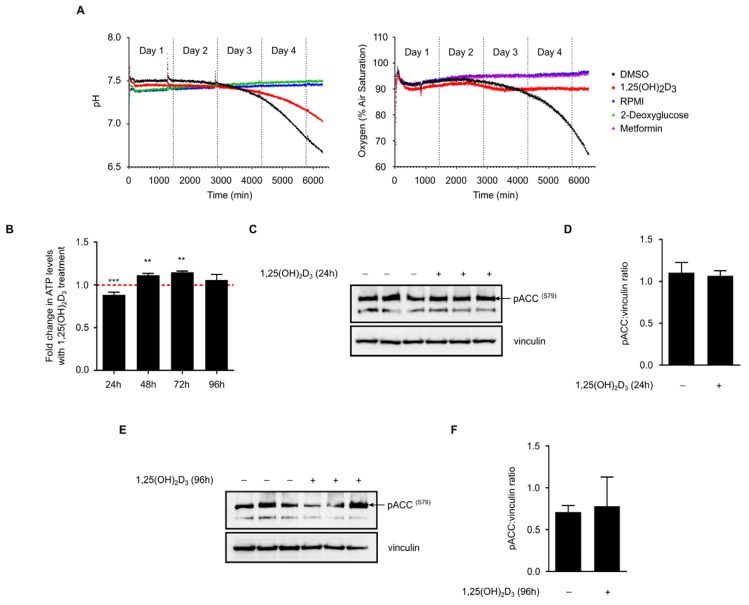
1,25(OH)_2_D_3_ does not profoundly impact glucose metabolism but influences overall energy status. (**A**) On-line measurements of culture medium pH and amount of dissolved oxygen in response to different treatments over a time course of four days. Culture medium without cells (RPMI) was used as calibration reference for the measurement of both parameters. 2-deoxyglucose (10 mM) and metformin (2 mM) were used as additional controls for pH or oxygen measurements, respectively. 1,25(OH)_2_D_3_ treatment did not predominantly influence glucose metabolism in HL-60 cells. Differences between DMSO and 1,25(OH)_2_D_3_ treated cells observed during the fourth day of measurement could by large be attributed to the drastic inhibition of cell proliferation by 1,25(OH)_2_D_3_. Data presented are representative of two similar biological replicates; (**B**) ATP levels were significantly reduced with 1,25(OH)_2_D_3_ treatment in 24 h, but were elevated with treatment after 48 and 72 h. The dashed red line indicates baseline ATP level in DMSO-treated cells set to 1. Statistical comparisons are made between DMSO- and 1,25(OH)_2_D_3_-treated cells using a two-tailed Student’s *t*-test. *p*-Values less than or equal to 0.01 and 0.001, are depicted by ** and ***, respectively. Error bars ± SD; *n* = 3; (**C**,**D**) 24 h treatment of HL-60 cells with 1,25(OH)_2_D_3_ did not significantly influence phosphorylation of ACC ^(S79)^; immunoblot and densitometric analysis. “+” and “−” denote the presence or absence of the indicated molecule/treatment, respectively; (**E**,**F**) Similar to 24 h-treated cells, a 96 h treatment did not profoundly influence ACC ^(S79)^ phosphorylation. Error bars ± SEM; *n* = 3.

To address this possibility, HL-60 cells were treated with either DMSO or 1,25(OH)_2_D_3_ and the pH as well as levels of dissolved oxygen in the culture medium were measured in real-time over the course of four days. No clear differences in the investigated parameters were observed after three days of treatment, however, at the beginning of the fourth day, both parameters were markedly reduced in DMSO-treated cells (Figure 5a). We attribute these differences to the strong reduction in cell number with 1,25(OH)_2_D_3_ treatment and not to actual metabolic reprogramming (Figure S2). 

On the other hand, intracellular ATP levels in 1,25(OH)_2_D_3_-treated HL-60 cells were found to be significantly reduced at 24 h and induced at the 48 and 72 h time points when compared to DMSO-treated cells (Figure 5b). In view of the lack of clear differences in glycolytic and oxygen consumption rates with 1,25(OH)_2_D_3_ treatment, we hypothesize that 1,25(OH)_2_D_3_ does not directly impact ATP production but rather utilization, possibly by inhibiting other ATP-consuming processes like fatty acid biosynthesis. We therefore postulate that preservation of ATP levels may constitute part of the mechanism through which 1,25(OH)_2_D_3_ regulates TXNIP expression. 

Recent studies have demonstrated that the intracellular energy sensor AMP-activated protein kinase (AMPK) is activated by 1,25(OH)_2_D_3_ in cancer cells [15,19]. AMPK is known to be activated by different intracellular cues, namely increases in either intracellular calcium levels or in the AMP:ATP ratio [20]. In response to these cues, AMPK activates energy-producing pathways, such as fatty acid beta-oxidation and glucose uptake, and inhibits energy-consuming ones, including fatty acid and protein biosynthesis [20]. Wu et al. [9] have recently shown that the activation of AMPK leads to TXNIP degradation and increased glucose uptake. We thus speculated that the regulation of TXNIP expression by 1,25(OH)_2_D_3_ might be partly explained by modulation of this signaling pathway. We investigated the phosphorylation status of serine 79 of the AMPK substrate acetyl CoA carboxylase (ACC), as a biomarker of AMPK signaling activity, in response to 24 and 96 h of treatment with 1,25(OH)_2_D_3_. Treatment was not found to significantly influence this pathway at either time point (Figure 5c–f). 

## 3. Discussion

Recent studies have shown that TXNIP plays pivotal roles in regulating glucose and redox homeostasis [6,10]. Additionally, it is currently being viewed as a putative tumor suppressor that is based on its ability to induce apoptosis in cancer cells on one hand, and its expression being down-regulated/silenced in tumors on the other [10,11]. Furthermore, it has been demonstrated that the loss of TXNIP increases the predisposition to hepatocellular carcinoma [21]; therefore, reactivating/inducing TXNIP expression is thought to be beneficial to anti-cancer therapy. 

**Figure 6:**
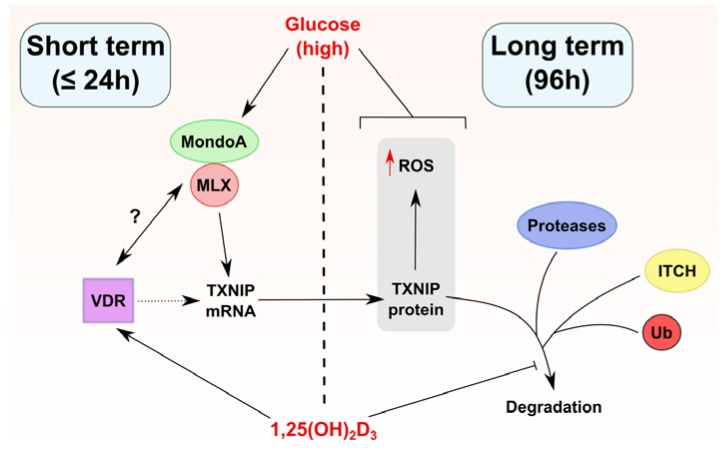
Proposed model of TXNIP regulation by 1,25(OH)_2_D_3_ in HL-60 cells. 1,25(OH)_2_D_3_ appears to regulate TXNIP expression through multiple mechanisms depending on the duration of treatment (long and short term treatment outcomes are separated in the figure by a dashed line). In short term treatments, 1,25(OH)_2_D_3_ induces TXNIP mRNA, but not protein levels, an effect that depends on the availability of glucose. This points toward the possibility that the VDR physically/functionally interacts with the transcriptional heterodimer MondoA/MLX, for example, via enhancing its binding to ChoRE on the TXNIP promoter, or via facilitating its recruitment/nuclear translocation. Such putative interactions are depicted in the figure by a question mark. On the other hand, in long term treatments, 1,25(OH)_2_D_3_ induces TXNIP protein expression as well as intracellular ROS levels, effects that are also glucose-dependent. Furthermore, long term treatment with 1,25(OH)_2_D_3_ influences TXNIP stability possibly through multi-modal regulation of TXNIP-degrading machinery, for example the E3 ligase ITCH, different proteases, or proteasomal subunits. Whether the TXNIP gene harbors VDRE is unclear, thus the vitamin D receptor (VDR) is linked to TXNIP regulation via a dotted line. A blunted arrow (t-bar) indicates inhibitory activity.

Although TXNIP was originally identified in HL-60 cells as the VDUP1 [4], reports on the ability of 1,25(OH)_2_D_3_ to induce its expression in cancer cells of diverse tissue origins are sparse, and have been largely limited to HL-60 cells. In our study presented here, we found no evidence for a direct link between vitamin D treatment and TXNIP levels. In fact, TXNIP levels are differentially regulated by 1,25(OH)_2_D_3_ in different cancer cell lines (Figure 1). Furthermore, induction of TXNIP in HL-60 cells on mRNA and protein levels by 1,25(OH)_2_D_3_ is glucose-dependent, unlike the regulation of the direct 1,25(OH)_2_D_3_ target gene *CYP24A1* (Figure 3). An overview of the described regulation of TXNIP by 1,25(OH)_2_D_3_ in HL-60 cells is presented in Figure 6. 

Several findings of the current report elicit a number of questions pertaining to the nature of TXNIP regulation by 1,25(OH)_2_D_3_, such as whether the *TXNIP* gene is really a direct target of 1,25(OH)_2_D_3_, and, in instances where TXNIP expression is reduced by 1,25(OH)_2_D_3_, whether this is the result of direct VDR-mediated trans-repression or secondary/indirect effects. Stambolsky and coworkers [22] have demonstrated that mutant p53 alters the transcriptional activity of the VDR in response to 1,25(OH)_2_D_3_ treatment, converting the latter from an anti-cancer to a pro-survival agent. The authors demonstrated that in cell lines that are harboring mutant p53, such as MDA-MB-231 and SW480, the mRNA expression of TXNIP, a pro-apoptotic gene, was reduced in response to 1,25(OH)_2_D_3_ treatment [22]. While this may serve as an explanation for the non-canonical regulation of TXNIP by 1,25(OH)_2_D_3_ in certain scenarios, we posit that additional modes of regulation may exist since in cell lines with wild-type p53, such as LNCaP and MCF-7, we also observed a clear reduction in TXNIP expression in response to 1,25(OH)_2_D_3_, and in HL-60 cells, which lack p53 expression [23], 1,25(OH)_2_D_3_ induces TXNIP levels. 

TXNIP appears to be at the crossroads of various signaling molecules implicated in tumorigenesis and anti-cancer treatment, such as Myc [24], AMPK [9], and mTOR [25], making its regulation subject to diverse cellular processes. Based on our previous findings in prostate cancer cells [15], as well as findings of the current study on HL-60 cells, we postulate that several pathways influenced by 1,25(OH)_2_D_3_ may contribute to the observed TXNIP regulation, such as regulation of metabolism-associated signaling molecules, namely AMPK, as well as the modulation of protein stability/degradation.

Studies have shown that the role of AMPK in cancer is contextual, with both beneficial and detrimental outcomes associated with its activation [26]. While the inhibition of mTOR activity as well as activation by the tumor suppressor LKB1 serve as a basis for AMPK’s anti-tumor effects, induction of potentially pro-survival pathways like autophagy and glucose uptake have been proposed to challenge the molecule’s anti-cancer role [26]. It is therefore possible that TXNIP degradation upon AMPK activation, described by Wu et al. [9], may contribute to the latter’s pro-survival effects. Paradoxically, 1,25(OH)_2_D_3_ and its analogues have been shown to activate AMPK signaling in different cancer cell lines [15,19,27]. Assuming that 1,25(OH)_2_D_3_ is capable of inducing AMPK signaling in different tumors, it would be interesting to characterize whether the consequence of this activation is TXNIP degradation or induction (through increasing glucose uptake and subsequently glycolytic intermediates that are capable of driving TXNIP expression). In this study, we show that AMPK signaling is insignificantly affected by 1,25(OH)_2_D_3_ treatment (Figure 5c–f), suggesting no or only minimal involvement of this pathway in regulating TXNIP expression by 1,25(OH)_2_D_3_ in this cellular context. Furthermore, we did not observe clear differences in glucose metabolism of HL-60 cells in response to 1,25(OH)_2_D_3_ (Figure 5a). Despite this, our results demonstrate a clear induction in ATP levels with treatment at certain time points (Figure 5b), which, as previously mentioned, may drive TXNIP expression through increasing the availability of glycolytic intermediates. 

Another interesting finding observed in this study is the lack of reduction in TXNIP levels upon addition of CHX to HL-60 cells treated with 1,25(OH)_2_D_3_ for 96 h (Figure 4c,d). This observation supports the possibility that 1,25(OH)_2_D_3_ influences TXNIP levels independent of direct transcriptional regulation. We explored the possibility that 1,25(OH)_2_D_3_ could lead to TXNIP stability through reducing the expression of the E3 ubiquitin ligase ITCH. Although ITCH mRNA levels were significantly reduced by 1,25(OH)_2_D_3_ after 24 and 96 h of treatment (Figure 4e), protein levels were found to be unaffected (Figure 4f–i). Results from others have illustrated the ability of 1,25(OH)_2_D_3_, and its analogues, to influence numerous players that are involved in regulating protein stability/degradation, including ubiquitin proteasome pathway (UPP) players, different proteases, as well as protease inhibitors [18]. For example, in colon cancer cells, 1,25(OH)_2_D_3_ treatment was found to mediate an overall repression of numerous genes encoding proteins belonging to the UPP [18]. Furthermore, in HL-60 cells, Shimbara et al. [17] showed that 1,25(OH)_2_D_3_ treatment reduced mRNA levels of different proteasome subunits. In view of this, we assume that in our setting, 1,25(OH)_2_D_3_ treatment reduced the expression of genes involved in TXNIP degradation besides ITCH. This could explain why 1,25(OH)_2_D_3_ treatment initially induced the expression of TXNIP at early time points (e.g., 24 h), but later stabilized its levels by preventing its degradation, and thus, a reduction in TXNIP levels by CHX treatment was not observed in 96 h 1,25(OH)_2_D_3_-treated HL-60 cells (Figure 4c,d). In support of this is the observed hastened and potentiated increase in TXNIP mRNA levels in HL-60 cells treated with 1,25(OH)_2_D_3_ and CHX, when compared to 1,25(OH)_2_D_3_ alone, described by Chen and DeLuca in their initial report [4]. Moreover, they also described an increase in TXNIP mRNA levels with CHX treatment alone [4], which altogether indicates that inhibition of de novo protein synthesis may reduce the expression of a TXNIP mRNA-degrading protein. 

## 4. Materials and Methods 

### 4.1. Cell Culture

The following cell lines were included in the study and were maintained in a standard tissue culture incubator set to 37 °C and 5% CO_2_: HL-60, U937, and Jurkat (hematological cancers); LNCaP, DU145, and PC3 (prostate cancer); HepG2, HLE, and SK-HEP-1 (liver cancer); AsPC-1, BxPC-3, and MIA PaCa-2 (pancreatic cancer); MDA-MB-231 and MCF-7 (breast cancer); HCT116 and HT-29 (colorectal cancer). Cell lines representing hematological cancers were cultured in RPMI 1640-GlutaMAX™ medium (Gibco, Darmstadt, Germany) supplemented with 10% FCS (*v*/*v*) (Gibco), and 1% penicillin/streptomycin (*v*/*v*) (Gibco). Other cell lines were cultured in Dulbecco’s Modified Eagle Medium (DMEM)-GlutaMAX™ (Gibco), supplemented with 10% FCS (*v*/*v*) (Gibco), 1% penicillin/streptomycin (*v*/*v*) (Gibco). Treatments with 1,25(OH)_2_D_3_ (Cayman Chemicals—Biomol GmbH, Hamburg, Germany) were performed in standard medium for different time points. 2-deoxyglucose (Fluka-Sigma-Aldrich, Steinheim, Germany), MG-132 (Sigma-Aldrich, Steinheim, Germany), CHX (Fluka-Sigma-Aldrich), metformin HCl (Sigma-Aldrich), and phloretin (Sigma-Aldrich) were used as indicated. For glucose deprivation experiments, RPMI 1640 without glucose medium (Gibco) was used. 

### 4.2. RNA Isolation, cDNA Synthesis, and RT-qPCR

HL-60 cells were treated with 1,25(OH)_2_D_3_ for various time points, after which total RNA was extracted using QIAzol lysis reagent (Qiagen, Hilden, Germany). The purity and concentration of RNA samples were determined using a NanoDrop 2000 UV-Vis Spectrophotometer (Thermo Scientific, Darmstadt, Germany). 500 ng of total RNA were used to synthesize cDNA using ProtoScript^®^ II first strand cDNA synthesis kit (New England Biolabs, Frankfurt am Main, Germany), following the manufacturer’s instructions. Subsequently, qPCR was performed using the real-time thermal cycler qTower (Analytik Jena AG, Jena, Germany) to quantify mRNA levels. The following forward (for) and reverse (rev) primers (Eurofins Genomics, Ebersberg, Germany) were used: TXNIP for: 5′-CGCCTCCTGCTTGAAACTAAC-3′, rev: 5′-AATATACGCCGCTGGTTACACT-3′; CYP24A1 for: 5′-TGGGGCTGGGAGTAATACTGA-3′, rev: 5′-GAACGCAATTTCATGGGAGGC-3′; ITCH for: 5′-5TCTAGTAGCTGTGGTCGGGG-3′, rev: 5′-CACAAGGCCACCGTGAAATG-3′ and vinculin (as reference gene) for: 5′-CAGTCAGACCCTTACTCAGTG-3′, rev: 5′-CAGCCTCATCGAAGGTAAGGA-3′*.* Reactions were performed using ready to use master mix LightCycler^®^ 480 SYBR Green I (Roche, Mannheim, Germany). 

### 4.3. Intracellular ROS and Glucose Uptake Measurements Using Flow Cytometry (FACS)

HL-60 cells were seeded at a density of 200,000 cells/well in 12 well-plates and were immediately treated with 1,25(OH)_2_D_3_. For intracellular ROS determination at the time points indicated, cells were washed once with PBS (Gibco), incubated with 30 µM dihydroethidium (Biomol GmbH) for 15 min, harvested, and re-suspended in 500 µL PBS for FACS analysis.

For glucose uptake measurements, 50 µM of the fluorescently labeled glucose analog 2-[*N*-(7-nitrobenz-2-oxa-1,3-diazol-4-yl) amino]-2-deoxy-d-glucose (2-NBDG) (Cayman Chemicals—Biomol GmbH) was added to the culture medium 1 h before the treatment period was over, as previously described, with minor modifications [15,28]. Cells were subsequently harvested and re-suspended in 500 µL PBS for FACS analysis. FACS analysis was performed using the FACSCalibur instrument (Becton Dickinson, Franklin Lakes, NJ, USA) and the CellQuest™ software (Becton Dickinson). 

### 4.4. Determination of Glucose in Medium Using the Glucose Oxidase (GOx) Assay

The assay was performed, as previously described [15]. The GOx enzyme mix was prepared using the following constituents: 50 mg/L GOx (Sigma-Aldrich), 250 mM Tris pH 8.0, 40 mg/L HRP (Sigma-Aldrich), and 100 mg/L *O*-dianisidine (Sigma-Aldrich). 10 µL of the medium supernatant of DMSO- and 1,25(OH)_2_D_3_-treated cells (initial seeding 20,000 cells/well in 500 µL medium in a 24-well plate) were collected at different time points, and were diluted in water 10 times. 240 µL of the enzyme mix were added to 5 µL of each sample or standard, and incubated for 1 h at room temperature. Absorbance at 450 nm was measured using a Tecan Ultra plate reader (Tecan, Crailsheim, Germany) and glucose concentration in samples was determined with a calibration curve obtained from reference absorbance values of six different glucose standards (concentrations 0.1–1.0 g/L). 

### 4.5. On-Line Measurements of Cellular Bioenergetics

OxoDish and HydroDish 24-well plates (PreSens Precision Sensing GmbH, Regensburg, Germany) were used for on-line measurements of dissolved oxygen and the pH of the medium, respectively, as previously described [29]. Changes in dissolved oxygen reflect oxygen consumption by respiration and hence mitochondrial activity, whereas changes in medium pH indicate lactate production from glycolytic metabolism. The plates contain fluorescence based sensors that were embedded at the bottom of each well, that can be read out continuously using dedicated SensorDish Readers (SDRs) that were placed inside a standard cell culture incubator. For the measurements, 20,000 cells/well were seeded in 1 mL/well medium in either Hydrodish or Oxoplate, and placed on an SDR inside the incubator. Measurements were started immediately and signals were recorded every 3 min. When signals had stabilized—approx. 20 min after initiation—measurements were paused, and plates were taken from the incubator to start treatment adding either DMSO, 1,25(OH)_2_D_3_, and designated controls (metformin or 2-deoxyglucose for oxygen and pH measurements, respectively). Plates were then placed back inside the incubator and measurements were continued. 

### 4.6. Determination of Cellular ATP

HL-60 cells were seeded at a density of 5000 cells/well in 100 µL medium in a black 96-well microplate with a clear bottom (Costar^®^, Corning Incorporated, New York NY, USA). Cells were subsequently treated with 1,25(OH)_2_D_3_ for different periods, after which 100 μL of the substrate solution provided with the ATPlite^TM^ 1 step kit (Perkin Elmer, Rodgau, Germany) were added to each well. The Tecan Ultra plate reader (Tecan) was used to measure luminescence signals kinetically. The obtained values were then normalized to cell count. 

### 4.7. Western Blotting

After treatment, cells were harvested, washed once with PBS, and lysed using 6 M urea buffer supplemented with a cocktail of protease and phosphatase inhibitors, namely aprotinin, leupeptin, pepstatin, PMSF, sodium orthovanadate, and sodium pyrophosphate. Protein content of samples was determined using Bradford reagent (Sigma-Aldrich). SDS-PAGE was subsequently performed to resolve the samples, and proteins were then transferred onto PVDF membranes (GE Healthcare, Munich, Germany). Membranes were washed once in TBS-Tween for 5 min, and then blocked for 1 h at room temperature using 5% non-fat dry milk in TBS/Tween. Membranes were then washed once in TBS/Tween for 5 min and were incubated overnight at 4 °C with the primary antibody. Anti-VDUP1 (TXNIP) antibody was purchased from MBL, whereas anti-β-actin and anti-vinculin antibodies were purchased from Santa Cruz Biotechnology. Anti-ITCH, anti-phospho-ACC ^(S79)^, as well as anti-mouse and rabbit IgG horseradish peroxidase (HRP)-linked antibodies were purchased from Cell signaling technologies. Western Lightning^TM^ Plus-ECl (Perkin Elmer) was utilized as HRP substrate. Target proteins were detected using the Fujifilm LAS-3000 imaging system. 

### 4.8. Statistical Analyses

GraphPad Prism and Microsoft Excel were used for statistical analyses. ImageJ software was used for densitometric analysis. Two-tailed Student’s *t*-test was used to calculate significance in investigated paramters between 1,25(OH)_2_D_3_ and DMSO treatment. A *p*-value that was less than or equal to 0.05 was defined as statistically significant. In figures, *, ** and *** represent *p*-values less than or equal to 0.05, 0.01, and 0.001, respectively. Error bars ± SD unless otherwise stated.

## 5. Conclusions

This study describes the regulation of TXNIP expression by 1,25(OH)_2_D_3_ in different cancer models. The *TXNIP* gene is possibly not a primary target of 1,25(OH)_2_D_3_ since the canonical induction is not observed in all investigated cell lines, and, in HL-60 cells, the mRNA induction is transient and glucose-dependent. However, 1,25(OH)_2_D_3_ appears to induce a clear and sustained increase in TXNIP protein levels in the same cell line, possibly through regulating protein stabilizing mechanisms at late time points. Nonetheless, several questions remain unanswered with regards to regulation of the *TXNIP* gene by 1,25(OH)_2_D_3_, such as: (i) whether the VDR physically/functionally interacts with glucose-sensing transcriptional machinery, namely MondoA/MLX, and (ii) whether the *TXNIP* gene harbors VDRE and regulation by 1,25(OH)_2_D_3_ is subject to chromatin architecture. Additionally, the putative induction and stabilization of TXNIP levels by 1,25(OH)_2_D_3_ are potentially influenced by the myriad of cellular effects the molecule induces, such as regulation of glucose metabolism [15,30], protein degradation [18], and non-coding RNAs [31]. Moreover, the results question the role/importance of TXNIP in mediating 1,25(OH)_2_D_3_’s anti-cancer effects since in cell lines where TXNIP levels are reduced by 1,25(OH)_2_D_3_, such as LNCaP and MCF-7, treatment has been shown to induce profound anti-tumor actions [32,33,34]. On the other hand, the activation of TXNIP expression by 1,25(OH)_2_D_3_ in HL-60 cells described in this study coincides with a significant inhibition of cellular proliferation. Therefore, a better understanding of TXNIP’s context-dependent roles would shed light on its importance to calcitriol’s effects in tumor cells. 

## Supplementary Materials

Supplementary materials can be found at http://www.mdpi.com/1422-0067/19/3/796/s1.

## Abbreviations

1,25(OH)_2_D_3_	1,25-dihydroxyvitamin D_3_
ACC	Acetyl CoA carboxylase
AMPK	AMP-activated protein kinase
CAMKK	Calcium/calmodulin-dependent protein kinase kinase
ChoRE	Carbohydrate-response element
CHX	Cycloheximide
DMSO	Dimethyl sulfoxide
GOx	Glucose oxidase
MLX	Max-like protein X
ROS	Reactive oxygen species
TXNIP	Thioredoxin-interacting protein
Ub	Ubiquitin
UPP	Ubiquitin proteasome pathway
VDR	Vitamin D receptor
VDRE	Vitamin D response elements
VDUP1	Vitamin D_3_ upregulated protein 1
