# Supplementary material for: Expression of TXNIP in Cancer Cells and Regulation by 1,25(OH)2D3: Is It Really the Vitamin D3 Upregulated Protein?
Source: Int J Mol Sci. 2018 Mar 10;19(3):796. doi: 10.3390/ijms19030796 (PMC5877657; doi:10.3390/ijms19030796)
Supplement: Supplementary file 1 [file ijms-19-00796-s001.docx]

**

**

**Figure S1:** Glucose levels in medium of DMSO- and 1,25(OH)_2_D_3_-treated HL-60 cells assessed using the glucose oxidase assay. While glucose levels clearly drop in the medium of the reference DMSO-treated cells, glucose levels are not significantly reduced during the investigated period in 1,25(OH)_2_D_3_-treated HL-60 cells, most likely reflecting the decrease in proliferation upon 1,25(OH)_2_D_3_ treatment.

**
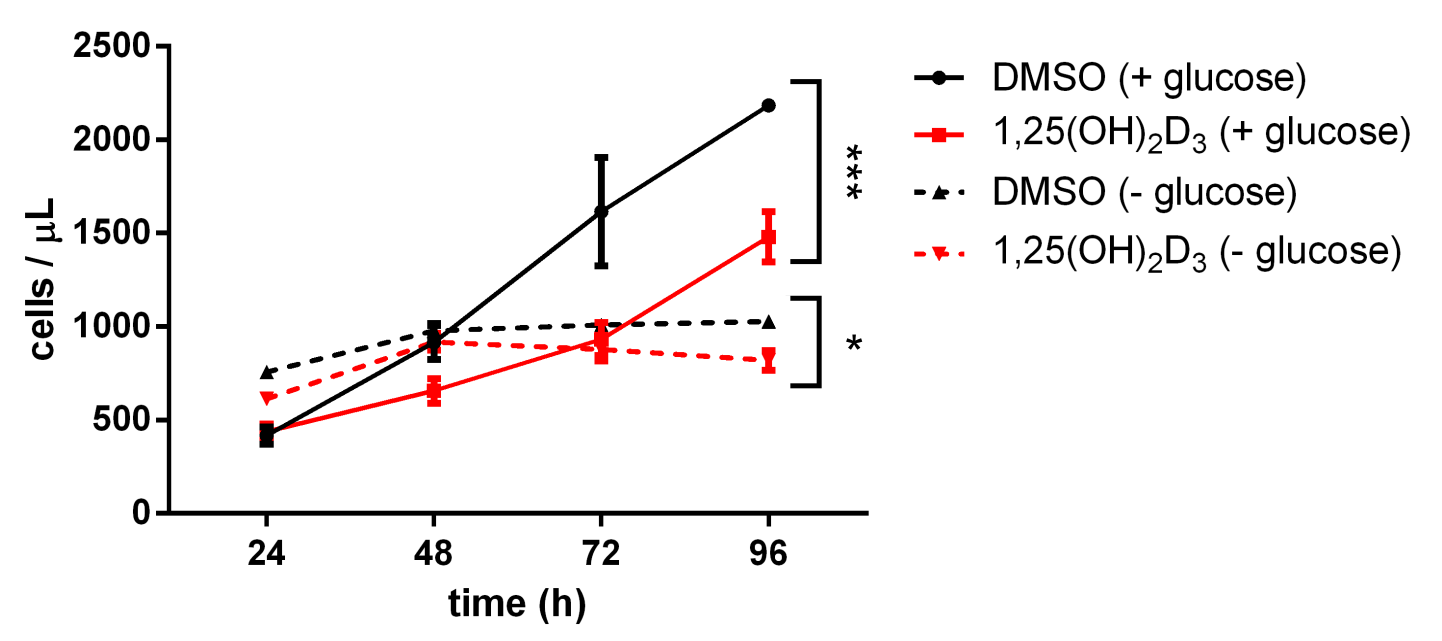
**

**Figure S2:** 1,25(OH)_2_D_3_ (100 nM) significantly reduces the proliferation of HL-60 cells cultured in RPMI medium with and without glucose. Statistical comparisons between DMSO- and 1,25(OH)_2_D_3_-treated cells are made using a two-tailed Student’s t-test. P-values less than or equal to 0.05, 0.01 and 0.001, are depicted by *, **, and ***, respectively.
